# Collaborative implementation of screening, brief intervention, and referral to treatment within the medical community of Blair County, PA

**DOI:** 10.1186/1940-0640-10-S2-O43

**Published:** 2015-09-24

**Authors:** Janice Pringle, Sherry Rickard-Aasen

**Affiliations:** 1 School of Pharmacy, Department of Pharmacy and Therapeutics, University of Pittsburgh, Pittsburgh, USA

## Background

This report provides an overview on the planning and implementation of Screening, Brief Intervention and Referral to Treatment (SBIRT) training programs in one community healthcare system so that it can be a model for other systems who wish to implement similar programs.

The goal was to reduce the impact of substance use disorders (SUD) on the criminal justice system and community by implementing SBIRT in local medical clinics to improve the early identification of and evidence-based intervention on SUD by the medical community.

## Material and methods

Two leading healthcare organizations in the community formed a committee that managed and spearheaded the SBIRT training program implementation. An Innovation Model used by the authors guided implementation. The committee completed organization assessments and business analyses for each training site. The committee and training sites became knowledgeable of SBIRT protocols, training program implementation, and infrastructure development. Once the program began, quality metrics were compiled and reviewed on a weekly basis.

## Results

Infusing SBIRT practices into clinics increased in difficulty as the complexity of the system increased, however, benefits were still obtained. Through the screening of over 280 new patients, training sites found that SBIRT exposed problematic substance use within their patient population that previously would have gone unnoticed. Identification presented opportunities to improve patient care. Knowledge and skills developed on screening, brief interventions; medical and psychiatric complications; and community D&A resources, were fundamental to implementation.

## Conclusions

The project revealed key factors relevant to the Innovation Model that related to successful implementation: champion preparedness; a highly specified protocol; a cultural shift signifying a reduction in negative stigma attached to working with patients with SUD; clear role definition for each member of the team; continuous activity tracking; and regular contact between physical and behavioral health providers and institutions.

